# *Helix pomatia* agglutinin bound to surface glycans of small extracellular vesicles *in-vitro* and* in-vivo* increases in early and late stage breast cancer

**DOI:** 10.1007/s12282-025-01724-4

**Published:** 2025-05-24

**Authors:** Jamie Cooper, Bethy Airstone, Ellie Beaman, Emanuela Carollo, Susan Ann Brooks, Ryan Charles Pink

**Affiliations:** School of Biological and Medical Sciences, Oxford Brookes University, Headington Campus, Oxford, OX3 0BP UK

**Keywords:** Extracellular vesicles, Cancer, Glycosylation, Biomarker, Diagnostics

## Abstract

**Background:**

Breast cancer is the most frequently diagnosed cancer and a leading cause of cancer-related mortality in women globally. Small extracellular vesicles (sEVs) play a crucial role in cell communication and cancer progression. This study aimed to investigate the glycosylation patterns of sEVs derived from breast epithelial cells and plasma samples from breast cancer patients, focusing on the presence of truncated O-linked glycans, such as the Tn antigen, using *Helix pomatia* agglutinin (HPA).

**Methods:**

Breast cancer cell lines were investigated for HPA lectin surface binding by confocal microscopy and flow cytometry. The sEVs of these were tested for surface HPA and tetraspanin binding using imaging-flow cytometry, single particle interferometry, and direct stochastical optical reconstruction microscopy. Plasma from healthy and stage II-IV breast cancer patients were tested by imaging-flow cytometry for HPA binding and analyzed for the source of HPA + EVs using 37 colocalised markers by multiplex flow cytometry .

**Results:**

Quantitative analysis revealed elevated HPA binding in sEVs from metastatic MCF-7 cells compared to that in non-metastatic BT-474 and immortalized healthy normal hTERT-HME1 cells, suggesting a correlation between HPA binding and metastatic potential. Analysis of sEVs revealed differential glycan presentation with CD81-positive sEVs from MCF-7 cells compared to CD63. In patient-derived plasma sEVs, HPA binding was significantly higher in patients with breast cancer than in healthy individuals, highlighting its potential as a biomarker for cancer detection.

**Conclusions:**

These findings highlight the complex glycosylation of sEVs and their potential early diagnostic utility in breast cancer for HPA positive sEVs.

**Supplementary Information:**

The online version contains supplementary material available at 10.1007/s12282-025-01724-4.

## Introduction

Breast cancer is the most frequently diagnosed cancer and the second leading cause of malignancy-related fatalities among women worldwide [14]. In 2020, approximately 2.3-million women were diagnosed with breast cancer, resulting in 685,000 deaths worldwide [50]. Despite advances in therapeutic interventions, a significant proportion of patients still face an incurable prognosis, breast cancer constitutes 1 in 8 cancer diagnoses and 1 in 6 cancer-related deaths, highlighting the substantial health burden breast cancer imposes on global healthcare systems [1]

Extracellular vesicles (EVs) are membrane-surrounded structures released by all cell types into the surrounding environment. These vesicles perform a wide array of biologic functions by mediating communication between cells, delivering bioactive cargo, and inducing phenotypic changes [11]. Their cargo includes proteins, lipids, metabolites, DNA, RNA, and non-coding RNA’s (miRNAs, tRNA, and rRNA) [10]. Among the various subtypes of EVs, small extracellular vesicles (sEVs), typically less than 200 nm in diameter, are especially significant. The uptake of sEVs has been shown to play a role in normal physiologic processes including immune system function, angiogenesis, and stress responses [56]. However, there is growing evidence of their role in the pathologic progression of several diseases, including cancer [37].

Glycans present on cargo carried by sEVs are often overlooked, despite growing evidence supporting their functional significance. Glycans are composed of linear or branched monosaccharide chains. They can be covalently attached to proteins, forming N-glycans when linked to asparagine residues, or O-glycans when linked to serine or threonine residues. The most common type of O-linked glycosylation begins with the attachment of a GalNAc monosaccharide to the protein, and is also known as O-GalNAc or ‘mucin-type’ glycosylation. Mucin-type O-glycosylation is one of the most diverse types of glycosylation and plays an essential role in the normal development, growth, and differentiation of cells and tissues [12, 42]. Abnormal mucin-type O-glycosylation is linked to numerous human diseases, including cancer [4]. Truncated O-glycans, including the Tn antigen (α-GalNAc-O-Ser/Thr), are commonly observed in cancer [49, 54]. Multiple studies have reported elevated levels of Tn antigen in approximately 90% of various human cancers, including breast, bladder, cervical, ovarian, colon, lung, gastric, and prostate cancers, whereas it is rarely present in normal adult cells [13, 46, 48].

Lectins are sugar-binding proteins of non-immune origin that agglutinate cells or precipitate glycoconjugates [21]. They possess at least one non-catalytic domain capable of reversibly binding to specific monosaccharides or oligosaccharides, enabling them to bind to the carbohydrate components in EVs [30]. The lectin from *Helix pomatia* (*Helix pomatia* agglutinin, HPA) has previously demonstrated the ability to detect glycans featuring α-GalNAc, including Tn antigen, and has received considerable attention in the search for changes in glycosylation patterns that are associated with cancer metastasis [47]. Brooks & Leathem, [7] conducted a retrospective study spanning 24 years, involving 373 breast cancer patients, of which 293 (79%) had tumours positive for binding of HPA and 80 (21%) had tumours negative for HPA binding. HPA binding correlated with the presence of lymph node metastases and was highly predictive of poor survival. Moreover, HPA binding has been demonstrated to predict poor survival in a variety of other types of cancers, including gastric [26], prostate [43], oesophageal [57], and lung [29] cancers.

Thus, it is well established that significant alterations in O-linked glycosylation is a feature of cancer progression, and is linked to metastasis and prognosis. Numerous studies have also highlighted the role of sEVs in mediating key cancer hallmarks by transferring bioactive molecules, including glycoconjugates, to recipient cells, thereby influencing cancer progression and metastasis. Despite this, the functional role of glycoconjugates in sEVs is comparatively underexplored. The study aims to investigate HPA binding in cell line and breast cancer blood sample derived sEVs to find a correlation in its diagnosis. Addressing this gap represents a promising avenue for research, as understanding the glycosylation patterns of sEVs could lead to novel diagnostic approaches for cancer. This can contribute to improved minimally invasive early and late stage cancer diagnosis and monitoring.

## Materials and methods

**Fig. 1:**
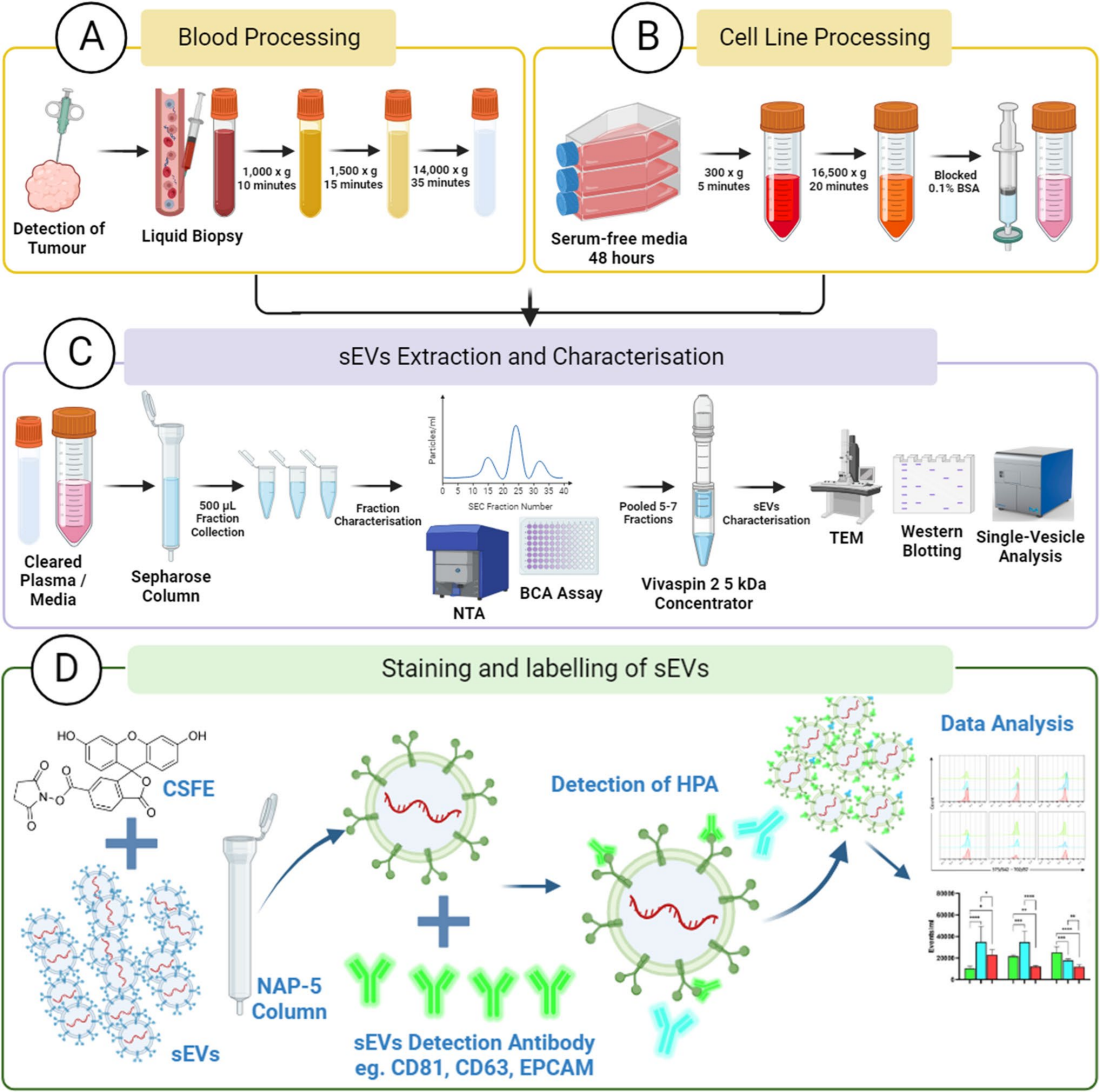
Workflow for the extraction, characterization and single-vesicle analysis of sEVs. **A** Blood samples from patients with known detected tumours were centrifuged to isolate plasma and further centrifuged to obtain cleared plasma samples. **B** Serum-free media from the cell line models were processed by centrifugation and filter-blocking with BSA. **C** The processed samples were subjected to SEC. Fractions eluted during this process were collected in 500 µL aliquots and evaluated by NTA and BCA assays to determine the specific fractions containing sEVs. Fractions containing high particle counts and low protein concentrations were pooled together and subjected to further characterization by TEM, western blotting, and single-vesicle analysis. **D** Staining and labeling of sEVs samples involved several key steps, including CFSE staining followed by SEC to remove unbound dye, treatment with the first detection antibody, and binding of HPA. The finalized labeled sEVs samples were then measured using the CellStream instrument

The present study aimed to investigate the binding profile of HPA sEVs derived from breast epithelial cells representing examples of healthy, primary cancer, and metastatic tumours, as well as plasma-enriched sEVs from healthy normal individuals and breast cancer patients at various stages (Fig. 1). This approach sought to elucidate the presence of the Tn antigen on these sEVs by HPA lectin binding and to assess its potential correlation with cancer progression and metastasis.

### Cell lines and culture of breast epithelial cells

All chemicals were purchased from Sigma-Aldrich (Merck), unless otherwise stated. The hTERT-HME-1 cell line was derived from healthy normal breast epithelium [15], the BT-474 cell line was derived from an invasive primary ductal breast cancer [31], and the MCF-7 cell line was derived from a pleural effusion from a metastatic breast cancer [44]. hTERT-HME-1 cells were maintained in DMEM/F12, 20 ng/mL epidermal growth factor (EGF) (PeproTech), 10 µg/mL insulin and 100 µg/mL hydrocortisone. BT-474 cells were cultured in RPMI-1640 medium and 2% v/v insulin. MCF-7 cells were grown in DMEM/F12. All cells were maintained additionally with 10% fetal bovine serum (Gibco) and 2 mM L-glutamine and cultured in a humidified atmosphere at 37 °C with 5% CO₂ and were negative for mycoplasma contamination.

### Extraction of small extracellular vesicles from breast epithelial cell lines

Cells were cultured to 50% confluence in T175 cm^2^ culture flasks, refreshed with serum-free medium, and cultured for a further 48 h. For sEV enrichment, the culture supernatant was centrifuged at 300 × g for 5 min, centrifuged again at 16,500 × g for 20 min at 4 °C, and the supernatant filtered through a 0.1% w/v BSA (Thermo Fisher Scientific) blocked 0.22- µm filter, then concentrated using VivaspinTM 20 concentrator columns (100 kDa, GE Healthcare), and centrifuged at 3,000 × g for 15 min at room temperature (RT) to 500μL. sEVs were enriched by size exclusion chromatography (SEC) using Econo-Pac^®^ chromatography columns (14 cm, Bio-Rad) loaded with 14 mL Sepharose gel filtration media (GE Healthcare, particle size 45 μm-165 μm), topped with a bed support and phosphate buffer saline pH 7.4 (PBS), settled overnight, and washed three times with 10 mL PBS. The sample was loaded, allowed to settle, and topped with 10 mL PBS. The flow-through was promptly collected in 500 μL fractions. sEV-enriched fractions 6–9 were pooled and further concentrated using Vivaspin 2 5 kDa concentrators (GE Healthcare) with centrifugation at 3000 × g for 15 min at RT. For short-term storage (up to 2 days), the samples were stored at 4 °C.

### Extraction of small extracellular vesicles from human plasma samples

Frozen human plasma (EDTA-K2) samples from normal ‘healthy’ donors and those with breast cancer (stage IIA M0 representing ‘non-metastatic sEVs’, and stage IV M1 representing ‘metastatic sEVs’) were purchased from Precision for Medicine Inc with ethical approval and consent under DHHS 45 CFR Part 46 and FDA 21 CFR Part 50/56 (Supplementary Table 1). Samples were defrosted on ice, and 500μL of plasma was centrifuged for 15 min at 1,500 × g at RT, the supernatant was centrifuged at 14,000 × g for 35 min at RT and transferred into a 1.5 mL tube. sEVs were isolated using SEC as described above. sEVs were characterized in accordance with the guidelines of the International Society for Extracellular Vesicles [52] (shown in Supplementary Fig. 1 A-G).

### Confocal laser scanning microscopy analysis of HPA labeling of breast epithelial cell lines

Confocal laser scanning microscopy was used to visualize and evaluate the binding of HPA lectin to breast epithelial cell lines. Coverslips with a diameter of 3 mm (Thermo Fisher Scientific) were sterilized with Industrial Methylated Spirit (IMS) and placed in 24-well plates (Corning). Cells were seeded into wells at 50,000 cells/ml and grown on coverslips to 70% confluence at 37 °C in a 5% CO_2_ atmosphere. Cells were washed three times with PBS and cell membranes stained using 1:1000 Cell Mask Deep Red (Thermo Fisher Scientific) for 5 min at 37 °C in the dark. The coverslips were washed three times with PBS to remove unbound dye. Cells were then incubated with HPA Alexa Fluor 588 conjugate (Thermo Fisher Scientific, L11271) at 7.5 μg/ml in 3% w/v bovine serum albumin/Tris buffered saline (BSA/TBS) (pH 7.4) for 10 min. Controls included a negative control (lectin omitted) and a sugar-specific competitive inhibition control (lectin was incubated with cells in the presence of 0.1 M GalNAc) to confirm specificity of lectin binding. The cells were then washed three times with PBS and fixed with 4% (v/v) paraformaldehyde in PBS at room temperature (RT) for 10 min. After three washes with PBS, coverslips were mounted onto glass slides with 200 μL Prolong Gold Antifade DAPI mounting medium (Thermo Fisher Scientific) and allowed to dry overnight at RT in the dark. Images were acquired using an LSM 800 confocal scanning laser microscope (Zeiss) with identical laser powers, detector gains, and offset settings. Cell samples were imaged in X-, Y-, Z-, and Z-stacks of 30–40 (0.2 μm) planes. Images were processed using FIJI software (v2.3.0), where the fluorescence signal in the channel corresponding to 488 nm excitation, indicative of HPA-lectin binding, was quantified. To analyze these images, each Z-stack was processed to select the membrane through membrane Cell-Mask signal thresholding using the method described by Huang & Wang, [24]. The mean fluorescence intensity (MFI) from the 488 nm excitation channel was measured and normalized to the area occupied by the cell mask membrane stain for each cell line.

### Flow cytometry analysis of HPA labeling of breast epithelial cell lines

Flow cytometry was used to quantify the binding of HPA to the surface of the breast cell lines. Cells were dissociated from the culture flasks using 5 mL of Accutase™ (StemCell Technologies) for 10 min at 37 °C. Cells were suspended at a cell density of 500,000 cells/ml and pelleted by centrifugation at 300 × g for 5 min, and then resuspended in 500 μL of 3% w/v BSA/TBS (pH 7.4) containing HPA Alexa Fluor 588 conjugate (Thermo Fisher Scientific, L11271) at 7.5 μg/ml for 10 min with previously mentioned controls. HPA labeling conditions were carefully optimized (Supplementary Fig. 2B), based on calculations of the stain index. The stain index was derived by subtracting the MFI of the background (unlabelled cells) from the MFI of the labeled cells and dividing this value by the standard deviation of the background MFI. For cell viability assessment, each sample was prepared by incubating it with 10 μL of a 1 mg/ml solution of propidium iodide for 10 min at room temperature. Samples were processed using a CytoFLEX flow cytometer (Beckman Coulter) equipped with 405-, 488, and 638 nm lasers. For analysis gating, single cells/singlets were isolated, and live cell populations were identified by side scatter area (SSC-A) and phycoerythrin area (PE-A) gates for negative propidium iodide staining. The results were analyzed using FlowJo v10.8.1 software quantifying the median values. Each experiment consisted of biologic triplicates, with three technical replicates.

### Nanoparticle tracking analysis

A ZetaView PMX 110 (Particle Metrix GmbH) instrument was used for nanoparticle tracking analysis (NTA) to assess the size distributions and concentrations of the pooled fractions containing sEVs. Calibration was performed using 100-nm polystyrene beads (Applied Microspheres) at a concentration ratio of 1:25,000. The sEVs were then diluted to a 1:1,000 ratio in a final volume of 1 mL of PBS for analysis. Data were acquired at RT using the settings: sensitivity 80, frame rate, 30 frames per second; shutter speed set to 100, minimum brightness 25, maximum pixel size 1,000, and minimum pixel size, 5.

### Western blotting for small extracellular vesicles markers

The expression of sEVs markers was determined by western blotting (Supplementary Table 2). Briefly, 50μL of cell lysates or 50μL of concentrated sEVs using 50μL 2X radio-immunoprecipitation assay buffer with Halt™ protease inhibitor cocktail (Thermo Fisher Scientific) and shaken for 20 min at 1000 rpm at 37 °C using a thermomixer. The protein concentration was assessed using a micro bicinchoninic acid assay (BCA) protein assay kit (Thermo Fisher Scientific). For cell and sEV samples, 5 μg total protein was mixed in 4 × Laemmli sample buffer (Bio-Rad), with or without 0.4 mM dithiothreitol and heated at 95 °C for 5 min. Samples were loaded onto 8–16% gradient polyacrylamide gels (Bio-Rad) with 5 µL precision plus protein standards (Bio-Rad) and subjected to electrophoresis at 125 V for 90 min in 10 × Tris/Glycine/SDS (Bio-Rad) running buffer. The separated proteins were transferred using a Trans-Blot Turbo RTA midi polyvinylidene difluoride transfer kit (Bio-Rad). Membranes were then blocked with 5% w/v skimmed milk powder (Marvel) solution in TBS 0.1% Tween (TBST) for 1 h at RT and incubated overnight at 4 °C with primary antibodies in 5% milk/TBST. Membranes were washed three times for 5 min in TBST, incubated for 1-h at RT with secondary antibodies in blocking buffer. The membranes were washed three times for five minutes in TBST and immersed in Clarity Max Western enhanced chemiluminescence substrate (Bio-Rad) for 5 min before imaging using a ChemiDoc MP Bio-Rad imaging system (Bio-Rad). The following primary antibodies were used: mouse antihuman CD63 1:1000 (10628D Thermo Fisher Scientific), rabbit antihuman syntenin-1 1:1000 (ab133267 Abcam), and rabbit anti-human cytochrome C 1:1000 (ab150422 Abcam). The following horse radish peroxidase (HRP)-conjugated secondary antibodies were used: goat anti-rabbit IgG-HRP 1:5000 (W4011, Promega) and goat anti-mouse IgG-HRP 1:20,000 (W4021, Promega).

### Transmission electron microscopy

Transmission electron microscopy (TEM) was employed to determine the morphological and size characteristics of sEVs. Carbon 300 mesh copper grids (TAAB Laboratories Equipment Ltd.) were glow-discharged for 20 sat 15 mA and 8 µl of sEVs were pipetted onto the grids and allowed to settle for 2 min at RT before the supernatant was blotted with filter paper. The grids were negatively stained with 10µL of 2% (w/v) uranyl acetate (Ladd Research) in distilled water for 10 s, blotted with filter paper, air-dried, and stored at RT until imaging. The samples were imaged using a Jeol JEM-1400 Flash TEM with a Gatan OneView 16-megapixel camera at 100 kV.

### Single-vesicle imaging flow cytometry

Single-vesicle imaging flow cytometry imaging of HPA-labeled sEVs was performed using an Amnis CellStream flow cytometer (Cytek/Luminex) equipped with 405, 488, 561, and 642 nm lasers and a charge-coupled device employing time-delay integration, with high-throughput and high-sensitivity detection. Initially, the CellStream was size-calibrated to resolve 30–150 nm sEVs using a vFC assay kit, composed of a synthetic vesicle size standard ‘Lipo100’ fluorescently labeled with vFRed as per instructions (Cellarcus Biosciences). All methods adhered to standardized sEV flow cytometry reporting guidelines (MIFlowCyt-EV) (Welsh *et al*., 2020). Controls included a negative (no cells), isotype (non-specific IgG antibody), sugar-specific (sugar-specific competitive inhibition, as described previously), detergent lysis (verifying sEV integrity), buffer-only (establishing baseline fluorescence), buffer with reagent (testing reagent alone), and unlabelled sample (assessing autofluorescence) control. Briefly, sEVs were stained with 40 µM carboxyfluorescein succinimidyl ester (CFSE) (ab145291 Abcam) for 2 h at 37 °C in the dark. Unbound CFSE stain was removed by passing the sample through a NAP-5 SEC column (GE Healthcare), as previously described [34]. Eluted sEVs were labeled for 1 h at 37 °C in the dark with a fluorescently labeled antibody, either Phycoerythrin (PE) labeled anti-CD81 (349,505 BioLegend) or anti-CD63 (353,003 BioLegend) for probing cell line-derived sEVs. An anti-EpCAM antibody (BioLegend, 324,205) was used to detect plasma-enriched sEVs (Supplementary Table 3). Subsequently, sEVs were labeled with Allophycocyanin (APC) labeled HPA (L32454, Thermo Fisher Scientific) for 1 h at 37 °C in the dark. The sEVs samples were diluted 1:20 in PBS and transferred to SuperPlate PCR 96 well plates (Thermo Fisher Scientific) then analyzed using the plate reader of the CellStream instrument with FSC turned off, SSC laser set to 5%, and all other lasers set to 100%, in the Small Particle Detection mode at a flow rate of 3.66 µL/min. HPA-positive sEVs were defined by gating on Lipo100 synthetic vesicles, followed by CFSE-stained EVs versus free-stain background and negative gating of the same label IgG isotype control (Supplementary Fig. 2 A). All antibodies and lectins were previously optimized by titrating MCF-7 sEVs until the maximum signal was achieved against the background (Supplementary Fig. 2 C–F). Two distinct analyses were performed, comprising MESF analysis to quantify the number of molecules on the surface of a sEV, and the assessment of the proportion of sEVs in a population that was positive for the presence of each detection antibody/lectin. To facilitate the comparison and normalization of the varying amounts of sEVs loaded in each sample, the events/ml for the final gated HPA-positive sEVs was calculated as a percentage of CFSE + events/ml. All results were analyzed using FlowJov10.8.1, and median values were converted into standardized MESF units.

### ExoView analysis

To further characterize the sEVs for the presence of common markers, a single-particle interferometric reflectance imaging sensor on the ExoView R100 platform (NanoView Biosciences) was used. The ExoView Human Tetraspanin kit with immobilized antibodies against CD41a, CD9, CD63, CD81 and IgG controls on silicon dioxide chips (NanoView Biosciences, Boston, MA) was used to capture sEVs. The sEVs samples were diluted in a manufacturer-supplied incubation solution and incubated overnight at RT on ExoView Human Tetraspanin Chips. After washing, the chips were incubated with the kit provided anti-CD9-AF647, anti-CD63-AF488, and anti-CD81-AF555 antibodies diluted in 5% bovine serum albumin for 1 h at RT. To identify the co-localisation of HPA binding with each tetraspanin, additional sEVs samples were also incubated with HPA (L32454 Thermo Fisher Scientific) alongside tetraspanin labelling. After rinsing and drying, image acquisition from each chip was carried out using the ExoView® R100 platform, and the data were analyzed using ExoViewer 3 (NanoView Biosciences) software. Fluorescent cut-offs were set relative to kit-provided IgG controls.

### Direct stochastical optical reconstruction microscopy (dSTORM) — *Oxford Nanoimaging (ONi)*

sEVs were processed for imaging by the ONi super-resolution nanoimager using the ONi EV Profiler kit v2.0, according to the manufacturer’s instructions. The tetraspanin trio capture solution was used to capture 10 µl of 1 × 10^9^ MCF-7 sEVs/ml over 75 min. The chips were incubated with kit provided “pan-EV” detection fluorescent solution for 10 min. Then incubated for 50 min with kit provided three-color tetraspanin fluorescently labeled antibody cocktail (anti-CD9, CD63, and CD81) detection and 8 µg/ml HPA attached Alexa Fluor 647 nm (L32454 Thermo Fisher Scientific in) W1 buffer. A final wash was performed, and imaging buffer was added to each lane immediately before imaging. A total of three fields of view (50uM x 85uM) were imaged at random and analyzed using the EV profiling app on the CODI software (23 June release), settings were: N rings 10, bin size 12, Max r 150, counting positive clusters with a minimum of five HPA fluorescent molecules, plus two tetraspanin trio and two Pan-EV molecules each.

### Multiplex bead-based flow cytometric analysis of EV surface proteins by MACSPlex Exosome Kit

Plasma-enriched sEVs were subjected to multiplex bead-based analysis by flow cytometry using the human MACSPlex Exosome Kit (Miltenyi Biotec, 130–108–813) to identify markers which co-localized with HPA binding, according to the manufacturer's instructions. The sEVs particle counts were determined by NTA, as described previously, to calculate the input amount of 1 × 10^9^ particles/ml sEVs by dilution with MACSPlex buffer. MACSPlex Exosome Capture Beads, containing 39 antibody-coated hard-dyed bead populations with includes controls, were added to each sample and incubated overnight on an orbital shaker at room temperature, protected from light. After incubation, the beads were washed by adding MACSPlex buffer to each sample, followed by centrifugation at 3000 × g for 5 min at RT. For HPA-positive sEV detection, HPA labeled with Alexa fluor 647 was added to each sample and incubated for 1 h on an orbital shaker at RT, protected from light. The HPA concentration was optimized using single-vesicle imaging flow cytometry (as described in Sect."Single-vesicle imaging flow cytometry"). The samples were washed with MACSPlex buffer and centrifuged at 3000 × g for 5 min at RT. After washing, the samples were resuspended in MACSPlex buffer, incubated for 15 min on an orbital shaker at room temperature, protected from light, and centrifuged again at 3000 × g for 5 min at RT. Finally, the samples were resuspended in MACSPlex buffer and transferred to a V-bottom 96-well plate for flow cytometry analysis. The samples were analyzed using a CytoFLEX flow cytometer (Beckman Coulter) for 10,000 single-bead events. Data analysis was performed using FlowJo v10.8.1 software. The background values of the MACSPlex buffer and isotype controls on MACSPlex exosome capture beads were subtracted from each sample. Values were normalized to the mean MFI of the expressed markers to determine the relative expression of each marker.

### Statistical analysis

GraphPad Prism v9 software was used to analyze all data which are presented as the mean ± SD. Normality was assessed using the Shapiro–Wilk test, and an appropriate parametric or non-parametric alternative with a multiple comparison test was then applied. To compare two sample means, a two-tailed paired t test was applied for the analysis of paired parametric data. For paired non-parametric data, the Wilcoxon matched-pair signed-rank test was performed. An unpaired t test with Welch’s correction was used for unpaired parametric data without assumptions of standard deviation. All statistical tests are outlined in the figure legends, and p-values are indicated by asterisks, denoting the following *p*-values: *p* < 0.05 *, *p* < 0.01 **, *p* < 0.001 ***, and *p* < 0.0001 ****.

## Results

### *Cell surface localisation of HPA binding in breast epithelial* c*ell lines*

**Fig. 2:**
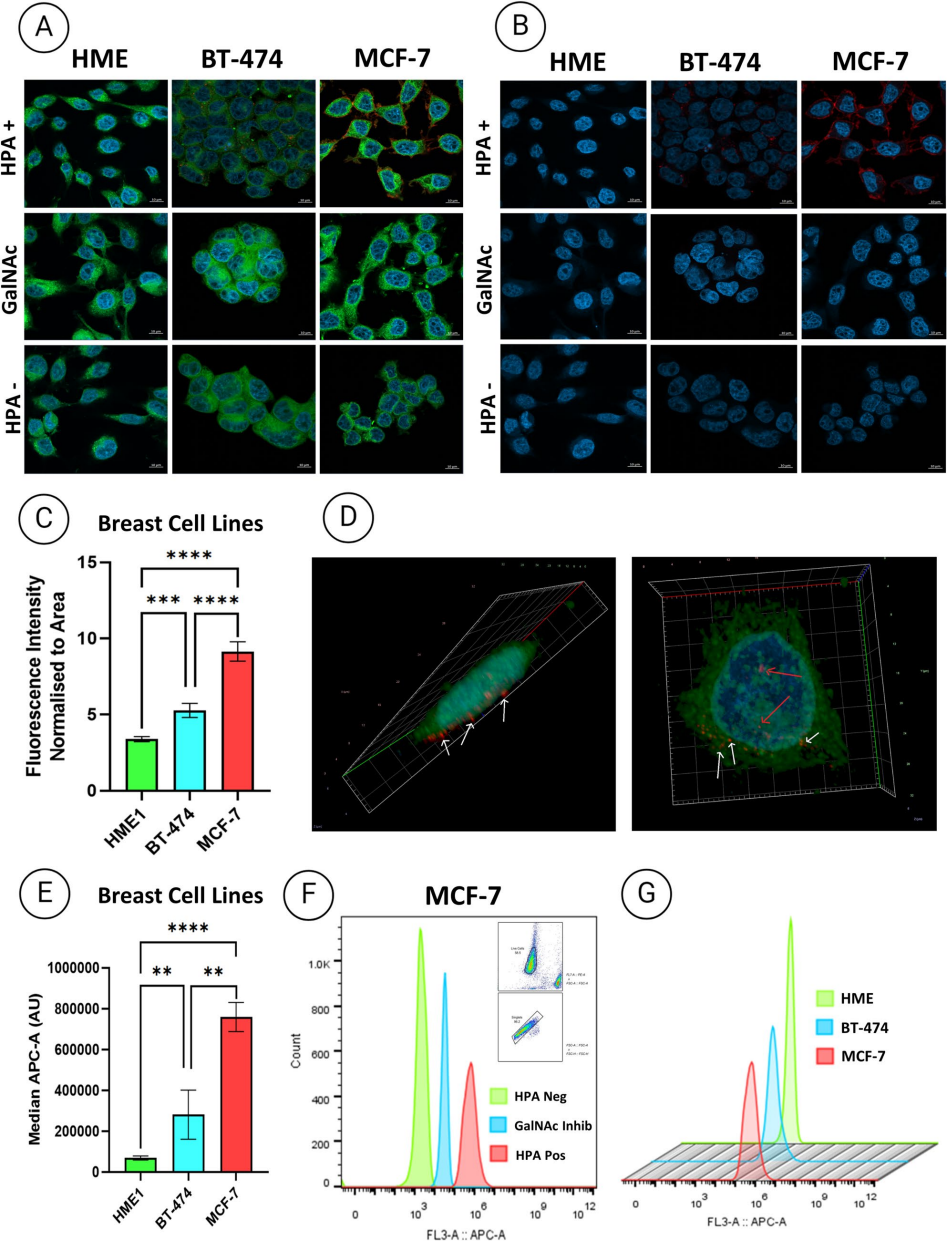
HPA glycosylation profiling of breast cell lines by confocal microscopy and flow cytometry. **A** Breast epithelial cell lines (hTERT-HME1, BT-474, and MCF-7) were probed with HPA lectin (red), CellMask™ to label the plasma membrane (green), and DAPI to label the nuclei (blue). The images show the HPA negative control (cells without HPA lectin), GalNAc inhibitor control (cells with HPA and sugar-specific control GalNAc), and HPA positive control (cells with HPA to identify the presence of GalNAc glycans). Scale bars = 10 µm. **B** The same images as in panel A, without plasma membrane overlay. **C** Quantification of fluorescence intensity normalized to the area occupied by the plasma membrane (green) for hTERT-HME1, BT-474, and MCF-7 cells. **D** An example of a Z-stacking image of a cell highlighting HPA lectin binding on the cell surface (red arrows indicate what appears to be internal labeling, but when the image is flipped, they appear to be external, represented by the white arrows). **E** Median fluorescence (AU) of breast cancer cell lines. Each experiment was performed in triplicate, with error bars representing the Stdev of the data. A t test followed by Welch’s correction was used for statistical analysis, and significance was set at *p < 0.05, **p < 0.01, ***p < 0.001, and ****p < 0.0001. **F** Histogram profiles of MCF-7 breast cell line example showing HPA binding alongside ancestral gating of live and single-cell exclusion with the HPA negative control, GalNAc inhibitor control, and HPA positive control. **G** Staggered flow cytometry histogram comparison of breast cancer cell lines for HPA positivity

To localise HPA lectin binding to the breast cell lines, lectin cytochemistry and Z-stack analysis of images captured using confocal laser scanning microscopy were performed (Fig. 2). Figure 2A–C shows specific HPA binding to breast cancer cells with increased intensity of labeling from hTERT-HME-1, derived from healthy normal breast epithelial cells, to BT-474, originally derived from a primary tumour, to MCF-7 originally derived from a breast cancer metastasis, with an average fold increase from non-cancer of 1.7 and 2.7, respectively (p < 0.0001). Z-stack analysis showed cell surface localisation of HPA labeling, suggesting the presence of surface O-GalNAc-glycans (Fig. 2D).

### Flow cytometry analysis of HPA lectin binding of breast epithelial cells

To confirm and quantify the binding of HPA to the breast cell lines, flow cytometry was performed (Fig. 2E-G) against controls (Fig. 2F). The resulting histograms, depicted in Fig. 2G, revealed a significant increase in HPA-binding cells in both cancer cell lines, BT-474 and MCF-7, compared with epithelial cells hTERT-HME1, derived from healthy breast epithelial cells, indicating the increased presence of surface O-GalNAc-glycans per cell. Interestingly, MCF-7 breast cancer cells, originally derived from breast cancer metastasis, exhibited an MFI average fold increase of 2 and 11 relative to BT-474, originally derived from a primary tumour, and hTERT-HME1 cells, derived from healthy breast epithelial cells, respectively (p < 0.0001) (Fig. 2E).

### Characterisation of breast epithelial cell line and patient plasma derived sEVs

Isolated sEVs were characterized and evaluated according to the MISEV guidelines (Supplementary Fig. 1 A-G) [52]. The sEVs were characterized for size and morphology using TEM, NTA, western blotting, and single-vesicle imaging flow cytometry to identify the presence of key sEV markers. TEM analysis revealed the detection of negatively stained, ‘cup-shaped’ particles within the size range of 30–150 nm for both sEV-enriched sample types matched by NTA size analysis. Western blot analysis revealed the efficiency of general SEC sEVs extraction by confirming the presence of key markers CD63 and syntenin-1 and negative control cytochrome C (a mitochondrial marker to show limited cell protein contamination carryover in the sEV sample).

### Comparative analysis of tetraspanins profiles of breast epithelial sEVs

**Fig. 3:**
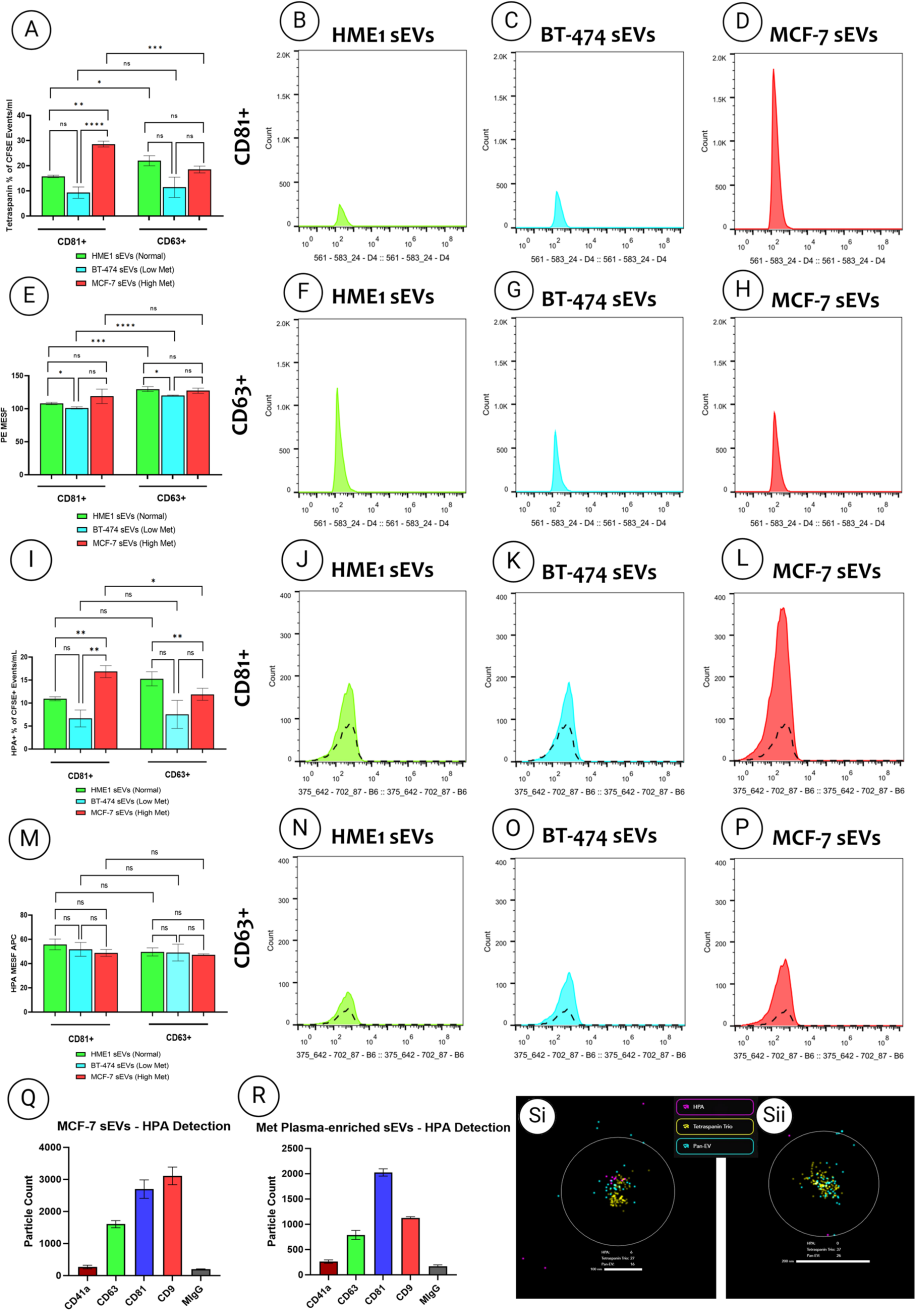
Imaging flow-cytometry, ExoView and super-resolution microscopy of tetraspanin and HPA binding in breast epithelial sEVs. **A** Quantification of CD81 + or CD63 + events/ml normalized to the percentage of CFSE events/ml in hTERT-HME1, BT-474, and MCF-7 sEVs. **B-D** Histogram profiles of breast sEVs showing CD81 fluorescence after gating. **E** PE MESF values of CD81 + or CD63 + for hTERT-HME1, BT-474, and MCF-7 sEVs with arbitrary units of MFI converted to standardized MESF units using vCal™ nanorainbow beads. **F–H** Histogram profiles of breast sEVs showing CD63 fluorescence after gating. **I** Quantification of HPA + events/ml normalized to the percentage of CFSE events/ml for hTERT-HME1, BT-474, and MCF-7 sEVs which are either CD81-postive or CD63-positive. **J-L** Histogram profiles of the breast sEVs after gating showing HPA lectin binding of CD81-positive sEVs fluorescence alongside the sugar-specific control GalNAc outlined in black. **M** APC MESF values of HPA + for hTERT-HME1, BT-474, and MCF-7 sEVs with arbitrary units of MFI converted to standardized MESF units using vCal.™ nanorainbow beads. **N-P** Histogram profiles of the breast sEVs after gating showing HPA lectin binding of CD63-positive sEVs fluorescence alongside the sugar-specific control GalNAc outlined in black. Each experiment consisted of biologic triplicates with three technical triplicates, and error bars indicate the Stdev. Normality of datasets was assessed using the Shapiro–Wilk test, and parametric or non-parametric tests were applied accordingly. For parametric t-tests, Welch’s correction was utilized, and for nonparametric t-tests, a Mann–Whitney test was used. Significance levels were set at *p < 0.05, **p < 0.01, ***p < 0.001, and ****p < 0.0001. **Q-R** Nanoview analysis example of MCF-7 and metastatic breast cancer patient plasma sEVs that are positive for HPA and tetraspanins. **S** Super-resolution microscopy example of MCF-7 sEVs labeled with HPA, tetraspanin trio **(i)** HPA positive **(ii)** HPA negative. HPA (magenta), tetraspanin trio (yellow) and Pan-EV (blue)

Single-vesicle imaging flow cytometry was performed to assess the CD81 and CD63 tetraspanin profiles of the breast epithelial sEVs (Fig. 3A-H). This approach enabled a detailed analysis of individual vesicles and the overall sEV population by measuring the MESF and concentration, respectively. The raw results, including the mean ± SD of CD81 and CD63 positivity, normalized to the CFSE-positive events (proxy of intact sEVs)/ml and MESF values, are given in Table 1. For sEVs from hTERT-HME1 cells derived from healthy breast epithelium, there was a 1.4-fold increase in the abundance of CD63 compared with CD81 (p < 0.05). In contrast, for sEVs from MCF-7 cells derived from breast cancer metastasis, there was a 1.5-fold higher abundance of CD81 than CD63 (p < 0.001). No significant difference was observed in the abundance of CD63 and CD81 in sEVs from BT-474 cells derived from primary cancer. Marked differences were noted in CD81 levels between sEVs derived from different cell lines, but such differences were not observed for CD63. Specifically, MCF-7 sEVs displayed 1.8-fold and 3.1-fold higher CD81 abundance than hTERT-HME1 (p < 0.01) and BT-474 sEVs (p < 0.0001), respectively. Conversely, there was no significant difference in CD81 levels between the hTERT-HME1 and BT-474 sEVs. MESF analysis revealed that, in both hTERT-HME1 and BT-474 sEVs, CD63 levels were 1.2-folder higher than those of CD81 in sEVs from the same cell types (hTERT-HME1: p < 0.001; BT-474: p < 0.0001). Interestingly, no significant differences in MESF values were observed between the two tetraspanins in MCF-7 sEVs. In addition, hTERT-HME1 sEVs exhibited 1.1-fold higher MESF values for CD81 and CD63 than those of BT-474 sEVs (p < 0.01). However, such differences were not observed in MCF-7 sEVs compared to other cells.

**Table 1:** Tetraspanin analysis of CD81 and CD63 on breast epithelial sEVs by single-vesicle flow cytometry

Cell Line	CD81 PE MESF (mean ± SD)	CD63 PE MESF (mean ± SD)	CD81 events/ml % of CFSE events/ml (mean ± SD)	CD63 events/ml % of CFSE events/ml (mean ± SD)	CD81 vs CD63 abundance	Vs HME sEVs	Vs MCF-7 sEVs
hTERT-HME1 sEVs	**108.16 ± 4.10**	**130.54 ± 8.96**	**16.033% ± 0.237**	**21.66% ± 4.96**	**Events/ml** * **MESF** ***		**Events/ml** CD81 = ** **CD63 = NS** **MESF** **CD81 = NS** **CD63 = NS**
BT-474 sEVs	**101.17 ± 3.62**	**119.43 ± 1.66**	**9.78% ± 5.09**	**10.41% ± 8.15**	**Events/ml** **NS** **MESF** ****	**Events/ml** **CD81 = NS** **CD63 = NS** **MESF** CD81 = * CD63 = *	**Events/ml** CD81 = **** **CD63 = NS** **MESF** **CD81 = NS** **CD63 = NS**
MCF-7 sEVs	**117.02 ± 26.42**	**129.02 ± 8.95**	**28.94% ± 2.59**	**18.16% ± 3.18**	**Events/ml** *** **MESF** **NS**	**Events/ml** CD81 = ** **CD63 = NS** **MESF** **CD81 = NS** **CD63 = NS**	

The asterisks (*) in the table denote significance levels: p < 0.001 *** and p < 0.0001 ****. *NS* not significant

### Tetraspanin subpopulation analysis of breast epithelial sEVs with fluorescently labeled HPA

Single-vesicle imaging flow cytometry was also used to assess HPA binding within the CD81 positive and CD63 positive tetraspanin populations of breast epithelial sEVs (Fig. 3I-P). The summary of the raw results, including the mean ± SD of HPA binding via CD81 or CD63 detection normalized to CFSE events/ml and MESF values, are shown in Table 2. Analysis of breast epithelial-derived sEVs revealed varying HPA-binding profiles associated with their tetraspanin composition. For MCF-7 sEVs, a pronounced increase in the number of EVs as a percentage of total sEV (by CFSE positivity) for HPA binding was observed co-bound to CD81-positive sEVs, showing a 1.4-fold higher level than that of CD63-positive sEVs (p < 0.05). For both hTERT-HME1 and BT-474 sEVs, there were no significant differences in HPA binding between the CD81-positive or CD63-postive sEVs. Interestingly, a significant increase in HPA binding was observed in CD81-positive MCF-7 sEVs, from cells derived from metastatic cancer, showing a 1.5-fold higher binding compared to sEVs from hTERT-HME1, cells derived from healthy breast epithelium, and a 2.5-fold increase compared to sEVs from BT-474, cells derived from primary cancer (p < 0.01).

**Table 2:** HPA lectin binding quantity (MESF) or percentage of total CFSE positive EVs split into CD81-positive and CD63-positive sEVs from breast and colorectal epithelial cells by single-vesicle flow cytometry

Cell Line sEVs	CD81-positive sEVs HPA MESF (mean ± SD)	CD63-positive sEVs HPA MESF (mean ± SD)	CD81-positive sEVs HPA events/ml % of CFSE events/ml (mean ± SD)	CD63-positive sEVs HPA events/ml % of CFSE events/ml (mean ± SD)	CD81-positive vs CD63-positive sEVs HPA	Vs HME sEVs	Vs MCF-7 sEVs
hTERT-HME1 sEVs	**56.62 ± 10.88**	**49.86 ± 7.19**	**10.84 ± 1.00**	**14.52 ± 3.91**	**Events/ml** **NS** **MESF** **NS**		**Events/ml** CD81 = ** CD63 = ** **MESF** **CD81 = NS** **CD63 = NS**
BT-474 sEVs	**52.21 ± 13.56**	**43.14 ± 17.16**	**5.85 ± 3.41**	**6.79 ± 6.36**	**Events/ml** **NS** **MESF** **NS**	**Events/ml** **CD81 = NS** **CD63 = NS** **MESF** **CD81 = NS** **CD63 = NS**	**Events/ml** CD81 = ** **CD63 = NS** **MESF** **CD81 = NS** **CD63 = NS**
MCF-7 sEVs	**49.44 ± 6.15**	**47.06 ± 1.64**	**16.82 ± 2.88**	**11.34 ± 3.28**	**Events/ml** * **MESF** **NS**	**Events/ml** CD81 = ** CD63 = ** **MESF** **CD81 = NS** **CD63 = NS**	

The asterisks (*) in the table denote significance levels: *p* < 0.05*, *NS*  not significant

To validate the presence of tetraspanins alongside HPA binding on the same particles, other single-particle analysis techniques, including ExoView analysis (Fig. 3Q-R) and ONi super resolution nanoimaging (Fig. 3Si-Sii), were employed. HPA labelling in conjunction with ExoView analysis confirmed the co-localisation of CD63, CD81, and CD9 in both MCF-7 cell-derived sEVs and plasma-enriched sEVs derived from stage IV breast cancer patients. With insignificant levels of CD41a. In MCF-7 cell-derived sEVs, HPA binding was most prominent on sEVs captured by CD9 and CD81, with reduced binding observed on CD63-captured sEVs. Conversely, in plasma-enriched sEVs from stage IV breast cancer patients, HPA binding was most abundant on CD81-captured sEVs, followed by CD9, with the least binding on CD63-captured sEVs. Moreover, ONi super resolution nanoimaging also confirmed the presence of the tetraspanin trio (yellow) and HPA (magenta) on MCF-7 sEVs.

### Diagnostic assessment of HPA binding of plasma-enriched sEVs derived from healthy individuals and breast cancer patients

**Fig. 4:**
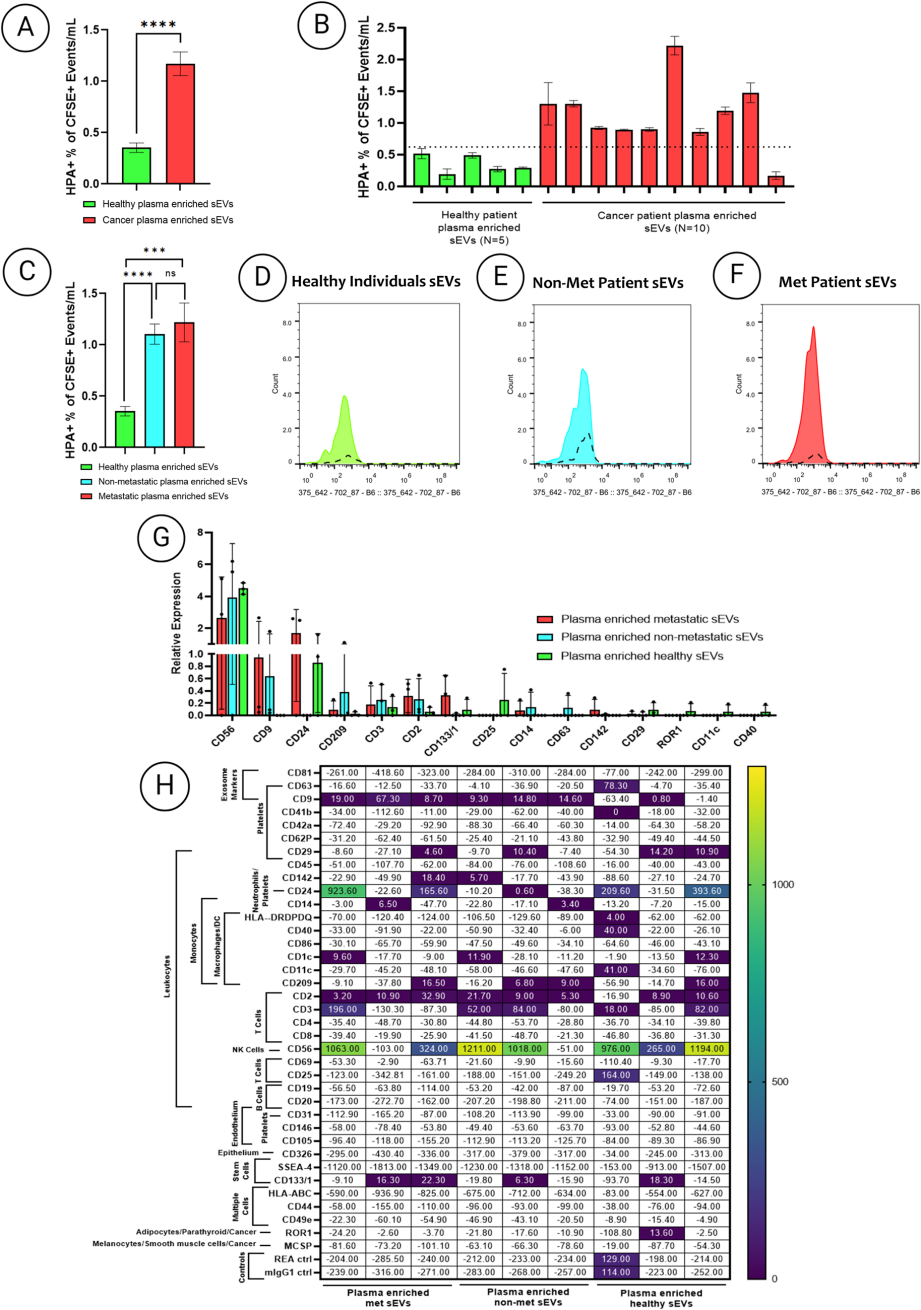
Imaging flow-cytometry and MACSPlex analysis of HPA+ plasma sEVs from healthy and breast cancer patients **A-C** Quantification of HPA+ events/ml normalized to the percentage of CFSE events/ml in plasma-enriched sEVs derived from healthy individuals and breast cancer patients. Healthy individuals (*n* = 5), non-metastatic breast cancer patients (*n* = 5) and metastatic breast cancer patients (*n* = 5). **D-F** CellStream histogram profiles of plasma-enriched sEVs after gating showing HPA fluorescence. Each experiment comprised biologic triplicates with three technical triplicates. **(G)** MACSPlex analysis of HPA EVs across common surface markers (*n* = 3 in each group). **H** MACSPlex analysis summary of plasma EV groups of HPA positive EVs, error bars represent the Stdev. All EVs were gated on EpCAM+ to focus on the epithelial EVs. The t test followed by Welch’s correction was used to determine the significance levels: *p < 0.05, **p < 0.01, ***p < 0.001, ****p < 0.0001

EpCAM was selected as a detection tool to specifically identify and analyze specifically epithelial cell-derived sEVs, which are uncommon in blood. Evaluation of HPA binding to plasma-enriched EpCAM-positive sEVs from healthy individuals and breast cancer patients are given in Fig. 4A-F. The primary data, including the mean ± SD of HPA-bound plasma-enriched EpCAM-positive sEVs normalized to CFSE events/ml and MESF values, are presented in Table 3. There was a 3.3-fold increase in HPA binding of plasma-enriched EpCAM-positive sEVs from cancer patients compared to those from healthy individuals overall (p < 0.0001). There was a 3.4-fold and 3.1-fold increase in HPA binding of EpCAM-positive plasma-enriched sEVs from individuals with metastatic and stage 2 non-metastatic breast cancer, respectively, compared to that seen in samples from healthy individuals (p < 0.0001 and p < 0.001, respectively).

**Table 3:** HPA lectin binding of EpCAM-positive plasma enriched sEVs derived from healthy individuals, non-metastatic and metastatic breast cancer patients

Plasma enriched sEVs	HPA APC MESF (mean ± SD)	HPA events/ml % of CFSE events/ml (mean ± SD)	Vs healthy sEVs	Vs metastatic sEVs
Healthy individuals sEVs	**63.748 ± 26.446**	**0.341 ± 0.168**		**Events/ml** *** **MESF** **NS**
Non-metastatic patient sEVs	**76.597 ± 9.989**	**1.106 ± 0.296**	**Events/ml** **** **MESF** **NS**	**Events/ml** **NS** **MESF** **NS**
Metastatic patient sEVs	**79.633 ± 27.297**	**1.153 ± 0.624**	**Events/ml** *** **MESF** **NS**	

The asterisks (*) in the table denote significance levels: *p* < 0.001 *** and *p* < 0.0001 ****, *NS* not significant

### MACSPlex analysis of HPA co-localisation in plasma-enriched sEVs derived from healthy individuals and breast cancer patients

A MACSPlex exosome kit was used to identify other common surface proteins co-bound with HPA on plasma-enriched sEVs from healthy individuals and patients with non-metastatic and metastatic breast cancer to provide insight into the source or role of these sEVs (Fig. 4G-H). HPA, serving as the detection probe, was used to identify glycoproteins co-localized with the patient plasma-enriched sEV samples across the 37 different sEV surface markers in the kit (Supplementary Table 4).

Initial comparisons of the common sEV markers on HPA-positive plasma-enriched sEVs derived from healthy individuals and patients with non-metastatic and metastatic breast cancer revealed little co-localisation with CD81 and CD63. Notably, HPA-positive plasma-enriched sEVs derived from patients with metastatic breast cancer exhibited MFI values of 19, 67.3, and 8.7, indicating significant co-localisation with CD9. In contrast, plasma-enriched HPA-positive sEVs derived from breast cancer patients with non-metastatic disease showed MFI values of 9.3, 14.8, and 14.6 for CD9 co-localisation.

The strongest co-localisation of HPA-positive plasma-enriched sEVs derived from each cohort was with CD56. Specifically, HPA +/CD56 + plasma-enriched sEVs derived from patients with metastatic breast cancer exhibited MFI values of 1063 and 324, respectively, with one patient showing no detectable MFI. Similarly, HPA +/CD56 + plasma-enriched sEVs derived from patients with non-metastatic breast cancer showed MFI values of 1211 and 1018, whereas one patient sample did not exhibit any measurable MFI value. HPA +/CD56 + plasma-enriched sEVs derived from healthy individuals had MFI values of 976, 265, and 1194, respectively.

Another marker which significantly co-localized in HPA-positive sEVs across different patient plasma samples was CD24. Specifically, HPA +/CD24 + plasma-enriched sEVs derived from patients with metastatic breast cancer exhibited MFI values of 923.6 and 165.6, while HPA +/CD24 + plasma-enriched sEVs derived from patients with non-metastatic breast cancer displayed minimal binding, with one patient sample showing an MFI value of 0.6. In contrast, HPA +/CD24 + plasma-enriched sEVs from healthy individuals showed MFI values of 209.6 and 393.6, with one sample also showing no binding.

Comparisons were also conducted to discern distinct binding patterns of HPA-positive plasma-enriched sEVs within specific cohorts, with the aim of identifying unique markers co-localized specifically between healthy and cancer groups, as well as between patients with non-metastatic and metastatic breast cancer. Interestingly, CD9 was significantly co-localized with breast cancer-associated HPA-positive plasma-enriched sEVs compared to sEVs derived from healthy individuals compared to patients with non-metastatic cancer (p < 0.05).

## Discussion

The binding of the lectin HPA to reveal truncated α-GalNAc-bearing O-linked glycans, including Tn antigen (α-GalNAc-O-Ser/Thr), is a well-established marker of metastatic competence in breast cancer (for example, [5, 7, 32, 33]). In this study, we employed a computational workflow for confocal laser scanning microscopy quantification to assess HPA binding in breast cancer cell lines, improving upon the traditional subjective visual scoring methods by reducing variability and bias. Our findings demonstrated that HPA labeling was localized at the cell surface, and, consistent with literature reports that HPA labelling of clinical tumour samples predicts metastasis and poor prognosis [5, 7, 32], we detected more intense HPA labeling in MCF-7 cells, originally derived from metastatic cancer [44] and of luminal A subtype and ER positive, PR positive and HER2 negative, than in BT-474, originally derived from primary breast cancer [31], of luminal B subtype and ER, PR and HER2 positive, and hTERT-HME1 cells, originally derived from normal breast epithelium [27], ER and HER2 negative (see Supplementary Table 5). Brooks *et al*., [6] have previously demonstrated that breast cancer cell lines, including MCF-7 and BT-474 cells, synthesize a heterogeneous range of glycoproteins featuring HPA-binding GalNAc-glycans that are common to clinical breast cancer samples. Flow cytometry provided an additional automated, quantitative assessment of HPA labelling, again, revealing more pronounced HPA binding in MCF-7 cells compared to BT-474 cells and hTERT-HME1 cells. To assess whether there was any correlation between ER/PR/HER2 presence, or histologic subtype of breast cancer, and HPA labeling, we collated the HPA labelling patterns across a number of breast cancer cell lines reported in the literature and did not find any correlation between HPA labeling and receptor status or histologic subtype (Supplementary Table 5).

sEVs were isolated from the breast cell lines and characterized for their tetraspanin composition and HPA-binding. Single-vesicle imaging flow cytometry was employed to assess tetraspanin composition, including MESF analysis to quantify surface molecules and assess the proportion of sEVs positive for each tetraspanin. We found a significantly higher abundance of CD81-positive sEVs derived from MCF-7 cells in comparison to CD63-positive sEVs. These findings align with the observations of Fan *et al.* [18], who similarly noted increased CD81 EV enrichment compared to CD63 on MCF-7 cells through western blot analysis. Interestingly, CD81 is involved in the regulation of cell migration and invasion, suggesting a role in cancer progression [55, 58]. Recent studies have highlighted the key role of CD81 in promoting cancer stemness and metastasis by interacting with CD44, a cell surface adhesion receptor which is highly expressed in many cancers [40]. Conversely, CD63-positive sEVs were more abundant in hTERT-HME1 and BT-474 cells. This aligns with the association of CD63 with less aggressive cancer phenotypes and its inverse correlation with metastasis [3, 9, 25, 38, 39].

Notably, MCF-7 CD81-positive sEVs showed increased HPA binding compared to CD63-positive sEVs derived from the same cell line, suggesting differential glycan presentation on sEVs based on their tetraspanin composition. Interestingly, other studies have suggested the presence of truncated mucin-type O-glycans, including the Tn antigen, on sEVs from cancers. For instance, HPA has been shown to bind sEVs derived from brain, cervical, and pancreatic cancer cells [16, 19]. The present study marks the first specific identification of an increased abundance of α-GalNAc glycans recognized by HPA on CD81-positive sEVs from MCF-7 cells compared to those derived from hTERT-HME1 and BT-474 cells. Moreover, analysis of the HPA binding of CD81-postive sEVs aligned with what is seen at the cellular level for HPA+ MCF-7 compared to hTERT-HME1, suggesting a potential parallel in glycan presentation between the cell surface and sEVs, but with nuanced specificity attributed to the tetraspanin composition. However, recent evidence has suggested a more refined relationship of specific glycoprotein presentations across the various cell types. For example, Nishida‐Aoki *et al. (* (36)identified distinct glycosylation patterns in sEVs derived from BMD2a cells, a brain metastatic subline of the human triple-negative breast cancer cell line, MDA-MB-231 revealing unique glycosylation profiles in BMD2a sEVs compared to those in their parental cells. Moreover, Gomes *et al.* [22] demonstrated that sEVs derived from OVMz ovarian carcinoma cells exhibit distinct glycosignatures, such as complex N-glycans and O-glycans, including T-antigen, differentiating them from whole-cell membranes. These findings are consistent with those of Escrevente *et al*. [17] who showed that sEVs from ovarian cancer cells were enriched in specific mannose-and sialic acid-containing glycoproteins. Collectively, these contrasting findings underscore the likelihood of a selective mechanism dictating the glycan composition of sEVs, distinct from that of their parental cells, revealing complex and highly specific processes of sEV glycosylation.

Further analysis of HPA binding in CD63-positive sEVs derived from hTERT-HME1 cells revealed a higher level of binding than that in CD63-positive sEVs derived from MCF-7 cells. Interestingly, when MCF-7 sEVs were characterized for CD63 by western blot analysis, smears were observed (Supplementary Fig. 1E), suggesting the presence of glycovariants. Despite evidence that CD63 is highly glycosylated, it may not be rich in α-GalNAc glycans recognized by HPA. A study conducted by Terävä *et al*. [51] determined that CD63 glycovariants in MCF-7 cells showed pronounced binding to *Ulex europaeus* agglutinin (UEA), which primarily recognizes Fuc(α1–2)Gal, but exhibited minimal interaction with *Vicia villosa* agglutinin (VVA), a lectin with carbohydrate specificity, for GalNAc, very similar to HPA. This observation may partly explain the reduced HPA detection in CD63-positive sEVs derived from MCF-7 cells. This suggests that the glycosylation profile of CD63 on these sEVs is skewed toward glycan structures that are not preferred substrates for HPA lectin.

As part of this study, we further analyzed the tetraspanin profiles of the sEVs derived from the cell lines and plasma samples and investigated whether the HPA-positive sEVs show a platelet marker using ExoView analysis, indicated by CD41a-positive sEVs. These results suggest that this is unlikely, as the CD41a signal was relatively low and close to the IgG control levels. Findings in this assay also suggests that increased co-localisation of HPA with CD9-positive sEVs at a lesser extent than other tetraspanins studied. It is well documented that cancer cells secrete a greater volume of EVs with altered composition than their non-malignant counterparts [2, 41]. In this study, we observed increased detection of HPA-positive sEVs in plasma-enriched sEVs derived from patients with breast cancer, including those at stages IIA and IV, compared to healthy individuals. These findings highlight the potential role of HPA along side EpCAM as a distinctive marker for epithelial cancer-derived sEVs, offering a means to differentiate malignant sEVs from those originating from non-malignant cells. Given the systemic nature of sEVs, their increased presence in the bloodstream may be influenced by the contribution of metastatic cells, potentially skewing the representation of sEVs. Alternatively, sEVs can be released from tumour cells to disrupt the extracellular matrix, which is characteristic of more metastatic behavior. However, it is important to note that the number of circulating tumour cells (CTCs) in the blood at any given time is typically very low and analytically unstable. Thus, it is more likely that the majority of sEVs originate from primary tumours or established metastases rather than directly from CTCs.

To further investigate the binding of HPA to plasma-enriched sEVs, MACSPlex analysis was performed. Given the highly heterogeneous nature of clinical plasma samples, this approach allowed for a more detailed assessment of the diverse sEV subpopulations, providing insights into the variability and complexity of their composition and glycosylation as revealed by HPA binding. Notably, HPA exhibited strong co-localisation with the CD56 marker in plasma-enriched sEVs derived from healthy individuals and breast cancer patients. Although CD56 is traditionally associated with natural killer cells, it is also expressed by a variety of other immune cells, including alpha beta T cells, gamma delta T cells, dendritic cells, and monocytes [53]. This co-localisation suggests that HPA may recognize specific glycan structures on sEVs associated with these immune cells, indicating a potential link between sEV glycosylation patterns and immune-related processes in both healthy and cancer conditions, leading to challenges in understanding the source of the specific systemic tissue-releasing HPA positivity of sEVs. However, further research is needed to confirm this hypothesis, as this association has not yet been extensively explored in sEV studies.

Another interesting finding was the observed increase in CD9 co-localisation in HPA-positive plasma-enriched sEVs derived from breast cancer patients compared to healthy individuals, though small, suggesting a potential avenue for diagnostic investigation. Although CD9 is commonly recognized as an sEV marker, its expression is widely associated with platelets [23, 28]. Moreover, the documented disparity in sEV release between cancer and non-malignant cells underscores the complex interplay between various components of cancer progression [2, 41]. In addition, elevated platelet counts have consistently been linked to disease-specific survival rates across various types of cancer, including breast cancer. For instance, in cancer patients with increased platelet counts, platelets play a multifaceted role in tumour progression [8]. They actively contribute to tumour growth and angiogenesis by predominantly releasing proangiogenic cytokines within the tumour microenvironment [20]. Platelets also facilitate tumour metastasis by shielding CTCs from shear stress and immune responses [35]. Hence, the integration of platelet-based biomarkers into emerging liquid biopsy approaches for cancer patients holds considerable promise for enhancing the diagnostic precision and predicting treatment outcomes. Given the potential diagnostic implications of HPA-positive sEVs and their co-localisation with CD9, further investigation of this interaction is warranted to explore its utility in enhancing breast cancer diagnosis. There are limitations with the study and its methods. The user needs to be highly skilled in sEVs extraction as the loss of pellets in various manual steps can be seen, hence why everything is compared back to a CFSE + sEV amount to show the EVs are present. Also due to the heterogeneity of sEVs and the presence of HPA binding on known blood groups then the reliance of EpCAM positive is relied upon to enrich the epithelial sEVs, making down stream assays more complex. Moreover, there is evidence of heterogeneity, and of prognostic significance, of EpCAM levels themselves in different breast cancer subtypes [45]. Also due to lack of space on ~ 100 nm EVs then dynamic range of HPA positivity is limited requiring strict thresholds, as seen with the cell work HPA does seem to increase with metastatic potential not seen in the sEVs. Also previous work has shown that HPA binding seems to be related to a metastatic phenotype of breast cancer, yet his paper shows its positivity at stages II with no clinical metastasis [7]. This can be seen as an opportunity with the growing interest in early micro-metastases seen in breast cancer. More experimental data is required to determine whether this phenomenon is seen in other cancer types, but it is indicative of potential value in diagnostics for early metastatic disease.

This study represents the first comprehensive investigation of HPA binding to sEVs derived from both breast epithelial cells and the plasma of healthy individuals and patients with breast cancer. Our findings confirmed the presence of HPA-binding glycans on these sEVs, aligning with the glycan profiles of the parental cells, particularly those influenced by the tetraspanin CD81. This consistency underscores the biologic significance of HPA-binding glycans in both cell-derived and circulating sEVs. Importantly, these results suggested that HPA binding could serve as a valuable marker for identifying tumour-associated sEVs, providing new insights into the glycosylation patterns of sEVs that may be critical in the context of breast cancer progression. Future studies are warranted to further elucidate these glycosylation profiles and their clinical implications, paving the way for novel, early, non-invasive diagnostic strategies in cancer.

## Supplementary Information

Below is the link to the electronic supplementary material.

Supplementary file1 (DOCX 1286 KB)

## Data Availability

The datasets used and/or analyzed during the current study are available from the corresponding author on reasonable request.
