# Supplementary material for: Helix pomatia agglutinin bound to surface glycans of small extracellular vesicles in-vitro and in-vivo increases in early and late stage breast cancer
Source: Breast Cancer. 2025 May 24;32(5):988–1005. doi: 10.1007/s12282-025-01724-4 (PMC12394263; doi:10.1007/s12282-025-01724-4)
Supplement: Supplementary file 1 — Supplementary file1 (DOCX 1286 KB) [file 12282_2025_1724_MOESM1_ESM.docx]

**Supplementary data**

***Table 1.*** ***Clinicopathological characteristics of human plasma samples (n=15)***

| **Samples** | **Age, years** | **Sex** | **Stage at Diagnosis** |
| --- | --- | --- | --- |
| **Healthy Individuals**  **(n=5)** | **44** | **F** | **-** |
|  | **34** | **M** | **-** |
|  | **47** | **M** | **-** |
|  | **60** | **F** | **-** |
|  | **64** | **M** | **-** |
| **Breast Cancer Patients**  **(n=10)** | **64** | **F** | **IIA** |
|  | **41** | **F** | **IIA** |
|  | **31** | **F** | **IIA** |
|  | **68** | **F** | **IIA** |
|  | **67** | **F** | **IV** |
|  | **77** | **F** | **IV** |
|  | **75** | **F** | **IV** |
|  | **60** | **F** | **IV** |
|  | **62** | **F** | **IV** |
|  | **54** | **F** | **IV** |


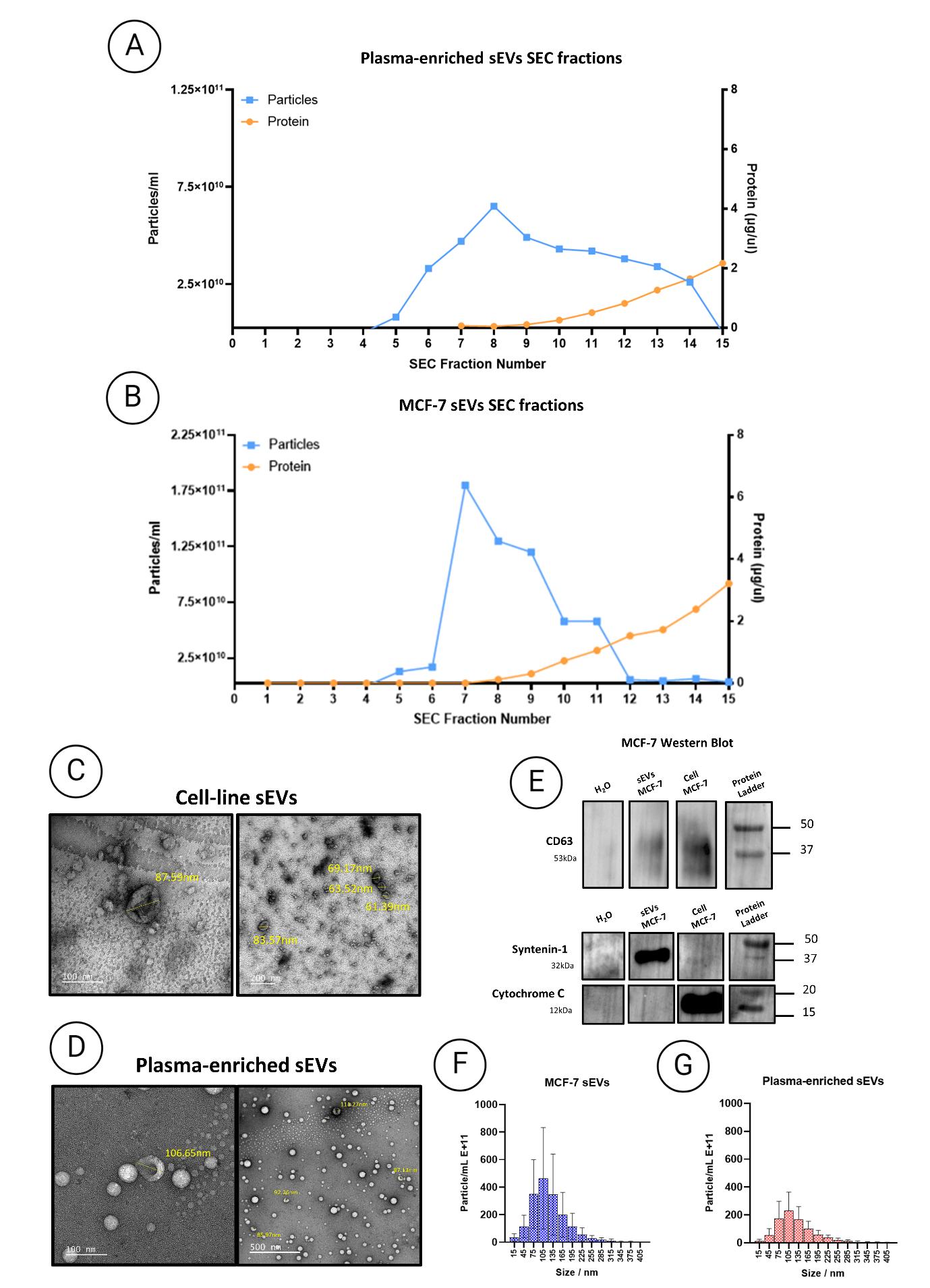


**Supplementary Figure 1. Characterisation of SEC fractions for the isolation of breast epithelial sEVs and plasma-enriched sEVs** **(A)** SEC fractions (500ul – obtained from 3 T175cm^3^ flasks) containing plasma-enriched sEVs were subjected to NTA and BCA analysis to determine the particle and protein concentrations to identify fractions which most likely contained sEVs. **(B)** MCF-7 sEVs **(C)** TEM analysis of MCF-7 sEVs from fractions 5, 6, and 7 pooled together. (Scale bars = 100 and 200 nm). **(D) P**lasma-enriched sEVs **(E)** Western blot of sEVs protein markers (CD63 and syntenin-1) and confirmation of sEVs lysates are clear cytochrome C marker indicating no mitochondria contaminates were present from dead cells. **(F)** NTA size characterisation of MCF-7 sEVs from pooled fractions 5, 6, and 7. **(G)** Plasma-enriched sEVs. The SEC fractioning experiment and western blotting consisted of one biological and technical replicate, whereas NTA each experiment consisted of three biological triplicates with three technical triplicates, and the error bars indicate the Stdev.


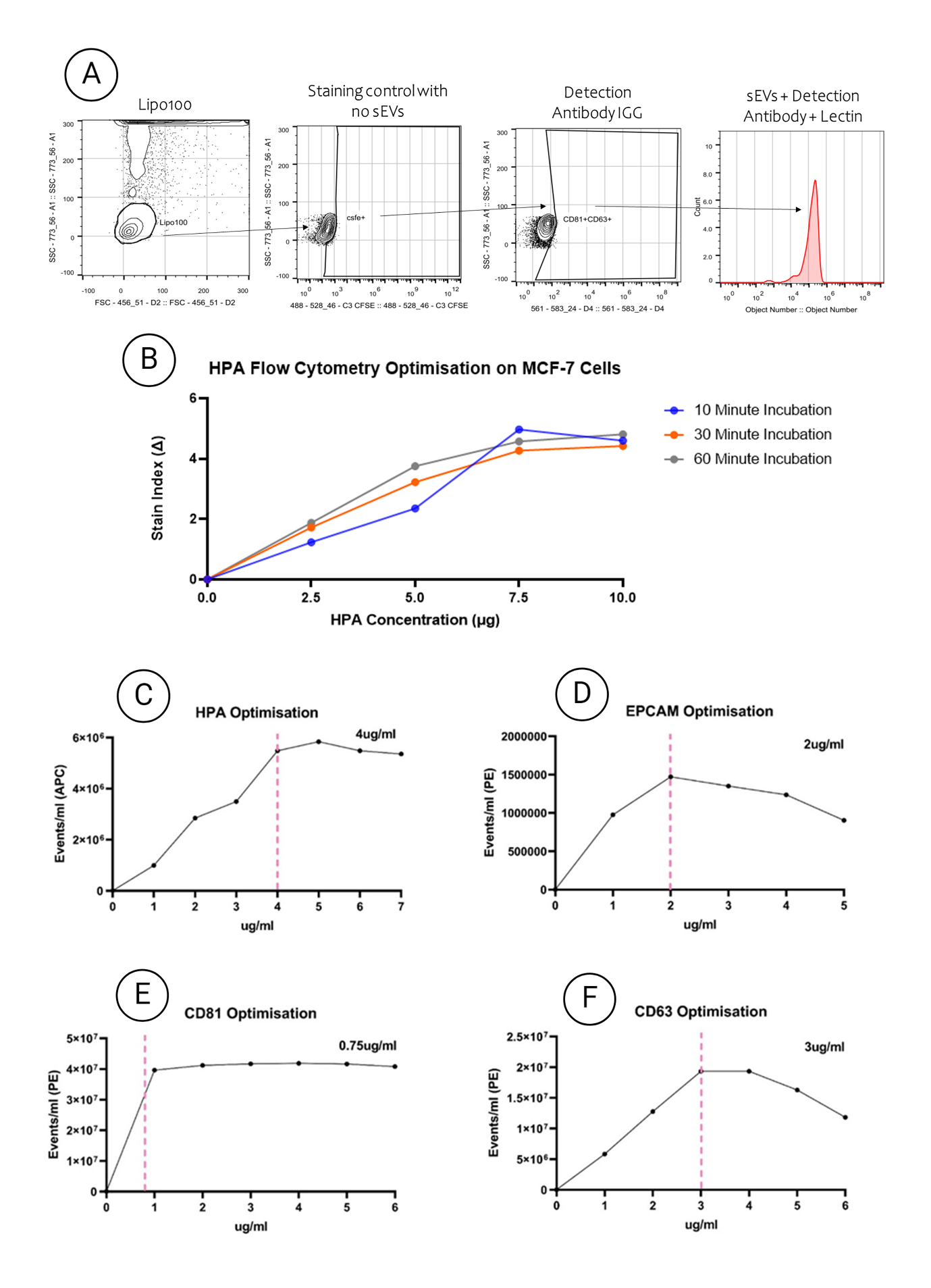


**Supplementary Figure 2. Cellular flow cytometry and Single-vesicle imaging flow cytometry optimisation (A)** Gating strategy adopted to identify sEVs. SSC and FSC gate to identify vFRed ^TM^ stained Lipo100 ^TM^ vesicle size standards used to size calibrate instrument. Control samples comprised of staining reagents used in experiment without sEVs to omit background signal. Isotype control of detection antibodies used in experiment to omit background signal. The quantified signal of interest after gating comprising of staining reagents used in experiment with sEVs. **(B)** Flow cytometry optimisation of HPA lectin using MCF-7 cells with concentrations ranging from 1 to 5 µg/ml at 10, 30, and 60-minute incubation times, employing a stain index calculation. **(C)** Optimisation of HPA Alexa Fluor 647 (4µg/ml) used for single-vesicle imaging flow cytometry analysis by determining the optimum events/ml achieved on MCF-7 sEVs. **(D)** EpCAM PE 561 optimisation (2µg/ml) **(E)** CD81 PE 561 optimisation 0.75µg/ml **(F)** CD63 PE 561 optimisation 3µg/ml.

**Table Sup2. Specifications of antibodies for western blotting for sEVs markers**

| **Target** | **Host** | **Dilution** | **Reducing Conditions** | **Manufacturer** |
| --- | --- | --- | --- | --- |
| **CD63** | **Mouse, monoclonal** | **1:1000** | **NO** | **ThermoFisher, 10628D** |
| **CD81** | **Mouse, monoclonal** | **1:1000** | **NO** | **Abcam, ab79559** |
| **Syntenin-1** | **Rabbit, monoclonal** | **1:1000** | **YES** | **Abcam, ab133267** |
| **Cytochrome C** | **Rabbit, monoclonal** | **1:1000** | **YES** | **Abcam, ab150422** |
| **ApoB** | **Rabbit, monoclonal** | **1:1000** | **NO** | **Abcam, ab139401** |
| **Rabbit IgG** | **Goat, Polyclonal** | **1:5000** | **N/A** | **Promega, W4011** |
| **Mouse IgG** | **Goat, Polyclonal** | **1:20000** | **N/A** | **Promega, W4021** |

**Table Sup3. Specifications of reagents used for single-vesicle flow cytometry**

| **Reagent** | **Conjugate** | **Isotype** | **Optimised Concentration** | **Manufacturer** |
| --- | --- | --- | --- | --- |
| ***Helix pomatia* agglutinin (HPA)** | **Alexa Fluor 647** | **-** | **4.0** **µg/ml** | **ThermoFisher, L32454** |
| **Carboxyfluorescein diacetate succinimidyl ester**  **(CFDA-SE)** | **-** | **-** | **40 µM** | **Abcam, ab145291** |
| **Epithelial cellular adhesion molecule (EpCAM)** | **PE 561** | **Mouse IgG2b, κ** | **2.0** **µg/ml** | **Biolegend, 324205** |
| **CD81** | **PE 561** | **Mouse IgG1, κ** | **0.75 µg/ml** | **Biolegend, 349505** |
| **CD63** | **PE 561** | **Mouse IgG1, κ** | **3.00** **µg/ml** | **Biolegend, 353003** |
| **Mouse IgG2b κ isotype Ctrl** | **PE 561** | **-** | **-** | **Biolegend, 401207** |
| **Mouse IgG1 κ isotype Ctrl** | **PE 561** | **-** | **-** | **Biolegend, 981804** |

**Table Sup4. The MACSPlex EV kit comprising of 37 surface epitopes that are present on sEVs plus two**

**isotype control beads**

| **Antibody** | **Isotype** | **Marker Groups** |
| --- | --- | --- |
| **CD81** | **Recombinant human IgG1** | **Exosome marker** |
| **CD63** | **Mouse IgG1k** | **Exosome marker** |
| **CD9** | **Mouse IgG1** | **Exosome and platelet markers** |
| **CD41b** | **Recombinant human IgG1** | **Platelet marker** |
| **CD42a** | **Recombinant human IgG1** | **Platelet marker** |
| **CD62P** | **Recombinant human IgG1** | **Platelet marker** |
| **CD29** | **Mouse IgG1k** | **Platelet and leukocyte marker** |
| **CD45** | **Mouse IgG2a** | **Platelet and leukocyte marker** |
| **CD142** | **Mouse IgG1k** | **Leukocyte marker** |
| **CD24** | **Mouse IgG1** | **Neutrophil, platelet and leukocyte marker** |
| **CD14** | **Mouse IgG2a** | **Monocyte and leukocyte marker** |
| **HLA--DRDPDQ** | **Recombinant human IgG1** | **Leukocyte, monocyte and macrophages marker** |
| **CD40** | **Mouse IgG1k** | **Leukocyte, monocyte and macrophages marker** |
| **CD86** | **Mouse IgG1** | **Leukocyte, monocyte and macrophages marker** |
| **CD1c** | **Mouse IgG2a** | **Leukocyte, monocyte and macrophages marker** |
| **CD11c** | **Mouse IgG2b** | **Leukocyte, monocyte and macrophages marker** |
| **CD209** | **Mouse IgG1** | **Leukocyte, monocyte and macrophages marker** |
| **CD2** | **Mouse IgG2b** | **T cell and leukocyte marker** |
| **CD3** | **Mouse IgG2a** | **T cell and leukocyte marker** |
| **CD4** | **Mouse IgG2a** | **T cell and leukocyte marker** |
| **CD8** | **Mouse IgG2a** | **T cell and leukocyte marker** |
| **CD56** | **Recombinant human IgG1** | **Natural killer cell and leukocyte marker** |
| **CD69** | **Mouse IgG1k** | **T cell and leukocyte marker** |
| **CD25** | **Mouse IgG1** | **T cell and leukocyte marker** |
| **CD19** | **Mouse IgG1** | **B cell and leukocyte marker** |
| **CD20** | **Mouse IgG1** | **B cell and leukocyte marker** |
| **CD31** | **Mouse IgG1** | **Platelet, endothelium and leukocyte marker** |
| **CD146** | **Mouse IgG1** | **Endothelium marker** |
| **CD105** | **Recombinant human IgG1** | **Endothelium marker** |
| **CD326** | **Mouse IgG1** | **Epithelium marker** |
| **SSEA-4** | **Recombinant human IgG1** | **Stem cell marker** |
| **CD133/1** | **Mouse IgG1** | **Stem cell marker** |
| **HLA-ABC** | **Recombinant human IgG1** | **Multiple cell marker** |
| **CD44** | **Mouse IgG1** | **Multiple cell marker** |
| **CD49e** | **Recombinant human IgG1** | **Multiple cell marker** |
| **ROR1** | **Mouse IgG1k** | **Adipocytes, parathyroid and cancer marker** |
| **MCSP** | **Mouse IgG1** | **Melanocytes, smooth muscle cell and cancer marker** |
| **REA ctrl** | **Recombinant human IgG1** | **Isotype control** |
| **mIgG1 ctrl** | **Mouse IgG1** | **Isotype control** |

*Table Sup5: Breast cancer cell line clinical subtypes and estrogen receptor (ER)/progesterone receptor (PR) and human epithelial receptor 2 (HER2) status (annotated from Dai et al., 2017; *hTERT-HME-1 stated in Kao et al., 2009). The cell lines are categorised into healthy/normal (N), luminal A (LA), luminal B (LB), Triple negative A (TNA) and Triple negative B (TNB). The derived disease type is coded as follows; N is 'healthy', AC is 'adenocarcinoma', B is 'benign tumour', IDC is 'invasive ductal carcinoma', MC is 'medullary carcinoma', SqC is 'squamous carcinoma'. This is matched to the HPA staining levels seen in this paper and other papers from the group. The top row contains the scale of HPA staining used in that paper. For both the 0-5 scale (Brooks et al., 2001) and the 0-3 scale (Schumacher et al., 1995) we suggest 2 or more to be a positive stain in this paper.*

Brooks, S. A., Hall, D. M., & Buley, I. (2001). GalNAc glycoprotein expression by breast cell lines, primary breast cancer and normal breast epithelial membrane. British Journal of Cancer, 85(7), 1014–1022. https://doi.org/10.1054/bjoc.2001.2028

Dai, X., Cheng, H., Bai, Z., & Li, J. (2017). Breast Cancer Cell Line Classification and Its Relevance with Breast Tumor Subtyping. Journal of Cancer, 8(16), 3131–3141.

Kao, J., Salari, K., Bocanegra, M., Choi, Y., Girard, L., Gandhi, J., Kwei, K.A., Hernandez-Boussard, T., Wang, P., Gazdar, A.F., Minna, J.D., Pollack, J.R. (2009). Molecular profiling of breast cancer cell lines defines relevant tumor models and provides a resource for cancer gene discovery. *PloS One,* 4(7), e6146. https://doi.org/10.1371/journal.pone.0006146

Schumacher, U., Adam, E., Brooks, S. A., & Leathem, A. J. (1995). Lectin-binding properties of human breast cancer cell lines and human milk with particular reference to Helix pomatia agglutinin. The journal of histochemistry and cytochemistry : official journal of the Histochemistry Society, 43(3), 275–281. <https://doi.org/10.1177/43.3.7868857>

Valentiner, U., Hall, D. M., Brooks, S. A., & Schumacher, U. (2005). HPA binding and metastasis formation of human breast cancer cell lines transplanted into severe combined immunodeficient (scid) mice. Cancer letters, 219(2), 233–242. https://doi-org.oxfordbrookes.idm.oclc.org/10.1016/j.canlet.2004.07.046
